# Effective Metabolic Carbon Utilization and Shoot-to-Root Partitioning Modulate Distinctive Yield in High Yielding Cassava Variety

**DOI:** 10.3389/fpls.2022.832304

**Published:** 2022-02-16

**Authors:** Porntip Chiewchankaset, Jittrawan Thaiprasit, Saowalak Kalapanulak, Tobias Wojciechowski, Patwira Boonjing, Treenut Saithong

**Affiliations:** ^1^Center for Agricultural Systems Biology (CASB), Systems Biology and Bioinformatics Research Group, Pilot Plant Development and Training Institute, King Mongkut’s University of Technology Thonburi (Bang Khun Thian), Bangkok, Thailand; ^2^Bioinformatics and Systems Biology Program, School of Bioresources and Technology, King Mongkut’s University of Technology Thonburi (Bang Khun Thian), Bangkok, Thailand; ^3^Institute of Biosciences and Geosciences (IBG-2): Plant Sciences, Forschungszentrum Jülich GmbH, Wilhelm-Johnen-Strasse, Jülich, Germany

**Keywords:** cassava, carbon utilization, carbon assimilation, carbon allocation, shoot-to-root carbon partitioning, crop yield, genetic potential, photosynthetic capacity

## Abstract

Increasing cassava production could mitigate one of the global food insecurity challenges by providing a sustainable food source. To improve the yield potential, physiological strategies (i.e., the photosynthetic efficiency, source-to-sink carbon partitioning, and intracellular carbon metabolism) can be applied in breeding to screen for superior genotypes. However, the influences of source-to-sink carbon partitioning and carbon metabolism on the storage root development of cassava are relatively little understood. We hypothesized that carbon partitioning and utilization vary modulating the distinctive storage root yields of high and low-yielding cassava varieties, represented in this study by varieties Kasetsart 50 (KU50) and Hanatee (HN), respectively. Plant growth, photosynthesis measurements, soluble sugars, and starch contents of individual tissues were analyzed at different developmental stages. Also, the diurnal patterns of starch accumulation and degradation in leaves were investigated through iodine staining. Despite a comparable photosynthetic rate, KU50 grew better and yielded greater storage roots than HN. Interestingly, both varieties differed in their carbon partitioning strategies. KU50 had a high photosynthetic capacity and was better efficient in converting photoassimilates to carbon substrates and allocating them to sink organs for their growth. In contrast, HN utilized the photoassimilates at a high metabolic cost, in terms of respiration, and inefficiently allocated carbon to stems rather than storage roots. These results highlighted that carbon assimilation and allocation are genetic potential characteristics of individual varieties, which in effect determine plant growth and storage root yield of cassava. The knowledge gained from this study sheds light on potential strategies for developing new high-yielding genotypes in cassava breeding programs.

## Introduction

Cassava (*Manihot esculenta* Crantz), a perennial crop plant, is recognized as an effective starch producer (El-Sharkawy, 2006). The starchy storage roots are the main diet of almost 1 billion people each year, highlighting the importance of cassava as a leading carbohydrate source for mankind, following only wheat, rice, and maize (Tonukari, 2004). With starch constituting 70%–90% of the storage roots dry matter (Nuwamanya et al., 2008), cassava provides more carbohydrate per unit cultivation area than other crop species (Nuwamanya et al., 2011). Additionally, cassava starch is used in versatile industrial production (Howeler et al., 2013) and consumption sectors. Annual production of cassava is required not only to provide food security for a growing world population but also to serve all sectors in downstream value chains.

Cassava was domesticated in the Amazon basin of Brazil and lowland Bolivia about 8,000–10,000 years ago (Brown et al., 2013), and its genetics has constantly been improved toward higher root yield ever since. Kasetsart 50 (KU50), a high-yielding variety bred in 1992 and grown widely across Thailand and Southeast Asia (Kittipadakul et al., 2017; Malik et al., 2020), possesses great agronomic traits, including high fresh storage root weight, dry matter, starch content, and harvest index (HI; Malik et al., 2020). By comparison, landraces like Hanatee (HN) and Munsuan have limited productivity with excellent cooking quality (Fu et al., 2014; Ceballos et al., 2020). Boonseng et al. (1999) reported 55.69 t ha^−1^ fresh root production, 23.6% starch content, and 0.45 HI for KU50, and 26.38 t ha^−1^ fresh root production, 15.6% starch content, and 0.34 HI for HN. The characteristic yields of KU50 and HN varieties were also reported in the recent studies, where KU50 showed 26.0–36.3 t ha^−1^ fresh root production, 24.4%–27.6% starch content, and 0.45–0.46 HI, and HN showed 12.1–22.5 t ha^−1^ fresh root production, 14.0%–32.4% starch content, and 0.34–0.45 HI (Santisopasri et al., 2001; Watananonta et al., 2006; Chiewchankaset et al., 2019; Chaengsee et al., 2020; Malik et al., 2020).

Cassava plant growth and storage root production depend on three main physiological characteristics, namely photosynthetic capacity, source-to-sink carbon partitioning, and intracellular carbon metabolism (De Souza et al., 2017; Aluko et al., 2021). The photosynthetic rate of cassava varies in a narrow range, 20–35 μmol CO_2_ m^−2^ s^−1^ in field conditions (El-Sharkawy et al., 1984; El-Sharkawy and Cock, 1990) and 13–24 μmol CO_2_ m^−2^ s^−1^ in greenhouses or growth chambers (Mahon et al., 1977; Edwards et al., 1990), in contrast with diverse yields observed in both systems. Previous studies show collective evidence of inconsistency between photosynthetic capability (μmol CO_2_ m^−2^ s^−1^) and final storage root yield, although a positive correlation has also been suggested (De Tafur et al., 1997; Long et al., 2006; El-Sharkawy and De Tafur, 2010). This complicated relationship is postulated to be mediated by the metabolic capability of individual genetic background. Due to the complexity of plant metabolism, research on carbon partitioning between source and sink tissues is limited, and almost all the studies are through indirect experiments, for example, by inferring from the growing biomass weight of individual plant parts and tracing the abundance of soluble sugar substrates in plant tissues (Luo and Huang, 2011; Duque and Setter, 2013; Hostettler, 2014; Li et al., 2016). Investigation of intracellular carbon conversion is often hampered by the impracticality of using current measurement methods for plants.

The influence of source-to-sink carbon partitioning and carbon metabolism on plant growth is a critical knowledge gap that hinders crop yield improvement. The processes dynamically change throughout plant development, from sprouting to final harvesting and are believed to affect the final root yield of cassava. Carbon utilization and allocation between source and sink organs have been proposed to be highly associated with the genetic potentials of individual varieties. Many cassava varieties grown in the same field under rain-fed conditions showed a wide variation in fresh storage root yield, root starch content, HI, and biomass accumulation (Boonseng et al., 1999; Santisopasri et al., 2001; Watananonta et al., 2006; Dixon et al., 2008; Chiewchankaset et al., 2019; Chaengsee et al., 2020; Malik et al., 2020). While the pattern of shoot-to-root carbon partitioning is predominantly affected by genetics, it can variably be altered by the surrounding environment depending on the varieties. Despite great insights gained from decades of efforts, little is known about carbon utilization, especially from photoassimilate translocation to root biomass production.

Recently, carbon metabolism in storage roots of cassava was comprehensively studied with the aid of a constraint-based metabolic model (Chiewchankaset et al., 2019). The study simulated carbon assimilation toward root biomass synthesis, described the metabolism underlying storage root growth rates of high- (KU50) and low-yielding (HN) varieties, and demonstrated the varietal differences in carbon utilization, proposed as one source of the yield distinction. In this study, we hypothesized that the carbon partitioning and metabolic processes determine the differences in the root yield between the two varieties. We studied the patterns of metabolic carbon utilization and shoot-to-root carbon partitioning in KU50 and HN cassava varieties for modulating effects on their distinct storage root yields.

## Materials and Methods

### Plant Materials and Cultivation

Kasetsart 50 (KU50) and Hanatee (HN) cassava varieties were grown in a controlled greenhouse environment (14/10 h of light/dark with <500 μE m^−2^ s^−1^ of natural light supported by mercury lamps (SON-T AGRO 400, Phillips, Netherlands), 29°C/24°C day/night, and 70% relative humidity on all days) during August 2016 to January 2017 at the Institute of Bio- and Geosciences Plant Sciences (IBG-2), Forschungszentrum, Jülich, Germany. KU50 is a widely grown commercial variety with high root yield and starch content, while HN is an edible low-yielding landrace. They were propagated by 10-cm long stem cuttings with at least two axillary buds. The individual stem cuttings were planted in commercial soil [containing natural organic (i.e., natural clay, peat moss, sod peat, coir, composted bark, and perlite), phosphorus (P_2_O_5_) 330 mg L^−1^, potassium (K_2_O) 480 mg L^−1^, nitrogen (N) 240 mg L^−1^, sulfur (S) 130 mg L^−1^, magnesium (Mg) 160 mg L^−1^, salt 2.5 g L^−1^, and adjust pH 5.8 with CaCl_2_ (Einheits Erde®)] in 25-cm diameter top, 19-cm diameter base, and 21-cm depth pots (c.a. 8,000 cm^3^ in volume) for 12 weeks, and then transferred to the 53-cm diameter top, 40-cm diameter base, and 43-cm depth pots (c.a. 75,000 cm^3^ in volume) to increase space for root growth. The plant positions were randomly rotated monthly during cultivation to ensure homogeneity of the microclimate to which each plant was exposed. Plants were watered twice a week with 1,000 ml of tap water per pot.

Fifteen plants were harvested every 4 weeks until 12 weeks after planting (WAP), and 12 plants at 15 and 20 WAP. The plant samples were separated into leaves, petioles, stems, stem cuttings, and total roots for growth measurement. The adventitious roots of cassava were also separated into fibrous roots (FR; <1-mm diameter) and early storage roots (ESR; ≥1-mm diameter) using the criteria modified from Keller (2014). To measure the starch and soluble sugar contents in diurnal conditions, the first fully expanded leaf on each plant, which located between the third leaf and the fifth leaf from the topmost of plants, was collected at 9:00 (morning), 12:00 (midday), and 18:00 (dusk/evening). Some separated plant parts (i.e., leaf, stem, FRs, and parenchyma tissues of ESRs) and the diurnal leaf samples were immediately frozen in liquid nitrogen and then freeze-dried at −55°C until the weight stabilized for analysis of sugar and starch contents later.

### Plant Physiology Measurement

The photosynthetic capability of KU50 and HN was examined based on measurements at the central lobe of the first fully expanded leaves of five plants taken at 11:00–12:30 every 4 weeks (i.e., 4, 8, 12, 15, and 20 WAP). Net photosynthetic rate (P_N_), transpiration rate (Tr), stomatal conductance (Gs), intercellular CO_2_ concentration (Ci), and the ratio between intercellular and ambient CO_2_ concentrations (Ci/Ca) were measured using a portable gas exchange system, infrared gas analyzer (LI-6400XT, Li-Cor Inc., Lincoln, NE, United States), equipped with a CO_2_ mixer to control the CO_2_ level in the chamber. The measurements were conducted with the following settings: 500 μmol s^−1^ air flow rate, 400 μmol CO_2_ mol^−1^ air CO_2_ concentration, 27°C leaf temperature, and 1,000 μmol photons m^−2^ s^−1^ light intensity. For respiration rate (R), it was measured using a portable gas exchange system according to the method described above unless the light intensity was set to zero. Chlorophyll fluorescence parameters were measured at the same lobe using Mini PAM-II Photosynthesis Yield Analyzer (Heinz Walz GmbH, Effeltrich, Germany) to measure steady-state fluorescence in the light-adapted state (*F*′) and the maximal fluorescence of the light-adapted state (*F*_m_′). The effective quantum yield of photosystem II [ΦPSII = (*F*_m_′ − *F*′)/*F*_m_′] and electron transport rate [ETR = ΦPSII × 0.84 × 0.5 × photosynthetically active radiation (PAR)] were calculated by the software of Mini PAM-II (Murchie and Lawson, 2013). The leaf chlorophyll content or leaf greenness index was measured at three different positions on the same lobe using the SPAD-502 leaf chlorophyll meter (Konica-Minolta, Japan), which measures the relative chlorophyll content per unit leaf surface area (Ling et al., 2011; Süß et al., 2015).

### Growth Measurements

Plant growth was examined by height, total leaf number (including attached and fallen senescent leaves), leaf area, and plant dry weight. Plant height was measured from the point of stem emergence to shoot apex. The attached leaves were counted from the first fully expanded leaf to the last photosynthetic leaf showing more than 50% greenness over its entire area, while the remaining leaves were counted as senescent. Total leaf area per plant (leaf lamina only) was measured using a leaf area meter (LI-3100C, Li-Cor Inc., Lincoln, NE, United States). Fresh and dry weights of all separated plant parts were determined. For dry weight measurement, all samples were oven-dried to a constant weight at 60°C. In addition to the destructive analysis, total root development in both cassava varieties was studied by MRI (van Dusschoten et al., 2016), a non-invasive method.

### Sugar and Starch Content Analysis

Sugar and starch contents in leaf, stem, FRs, and parenchyma tissues of ESRs were examined in three biological replicates using an enzymatic assay (Jones et al., 1977). Fifty milligrams of the ground freeze-dried sample were mixed with 80% ethanol and then incubated at 80°C for 15 min to extract soluble sugars. The extraction was repeatedly performed depending on the tissue type and plant age (Chow and Landhäusser, 2004). The soluble sugar concentration in aqueous extract was indirectly determined *via* the change of the reduced nicotinamide adenine dinucleotide phosphate (NADPH) during the enzymatic assay. The NADPH formation was measured through absorbance at 340 nm using a microplate spectrophotometer (SynergyTM 2, BioTek Instruments Inc., Winooski, VT, United States). The enzymatic assay started with mixing 20 μl of the extract with imidazole buffer, 36 mg ml^−1^ NADP^+^, 60 mg ml^−1^ ATP, and glucose-6-phosphate dehydrogenase. Subsequently, glucose, fructose, and sucrose concentrations were determined by absorbance measurement after adding hexokinase, phosphoglucoisomerase, and invertase, respectively. Each enzyme was added when the kinetic reaction reached saturation.

For starch measurement, the precipitate after the ethanolic extraction was mixed with 500 μl water before gelatinization in an autoclave at 135°C for 1 h. Next, the gelatinized starch was mixed with digestion buffer (50 mM Na-acetate pH 4.9, amyloglucosidase, and α-amylase) and incubated at 37°C for 16 h. After incubation, an aliquot of 20 μl was mixed with tris buffer, 36 mg ml^−1^ NADP^+^, 60 mg ml^−1^ ATP, glucose-6-phosphate dehydrogenase, and hexokinase. The total amount of glucose hydrolysate was determined by absorbance measurement at 340 nm with a microplate spectrophotometer (SynergyTM 2, BioTek Instruments Inc., Winooski, VT, United States).

### Chlorophyll Content Analysis

The extracted sample from the analysis of soluble sugars was adjusted with 95% ethanol in prior to the determination of leaf chlorophyll contents (i.e., total chlorophyll, chlorophyll a, and chlorophyll b) by using the UVIKON XL (BioTek Instruments, Winooski, VT, United States) spectrophotometer. The equations and specific absorption wavelength reported by Lichtenthaler (1987) were used, with 95% ethanol as blank.

### Iodine Staining

Diurnal patterns of starch accumulation and degradation in leaves were investigated through iodine staining. Stem cuttings of KU50 and HN cassava cultivars 10-cm in length were grown in pots [60-cm diameter top, 33-cm diameter base, and 45-cm depth pots (c.a. 79,000 cm^3^ in volume)] outdoor at the Center for Agricultural Systems Biology (CASB), King Mongkut’s University of Technology Thonburi (KMUTT), Thailand during June 2018 to December 2018. Treatments used were similar to the conditions mentioned above. Leaf apices of the first fully expanded leaf of 2-month-old cassava plants were collected at different times of the day: 6:00 (dawn), 12:00 (midday), 18:00 (dusk/evening), and 24:00 (midnight), to illustrate the phenomena of diurnal carbon accumulation and assimilation in cassava leaves. All leaf apices were submerged in 80% ethanol with vigorous shaking and incubated at 37°C for 8–12 h until all chlorophyll spots were removed. The staining was performed using 12.5% (v/v) iodine solution prepared from Lugol’s dye to visualize differentially accumulated starch (Hostettler et al., 2011). Excess iodine solution was removed by rinsing with distilled water. All pictures of iodine staining were taken by Fujifilm X-T2 under a light box to reduce shadow or light reflection.

### Statistical Analysis

All data were analyzed based on three biological replicates (mean ± SE), at least. Statistical testing was performed using a one-sided Student’s *t*-test with 95% confidence (*α* ≤ 0.05).

## Results

### Photosynthesis and Carbon Assimilation in Metabolism of KU50 and HN

**Figure 1:**
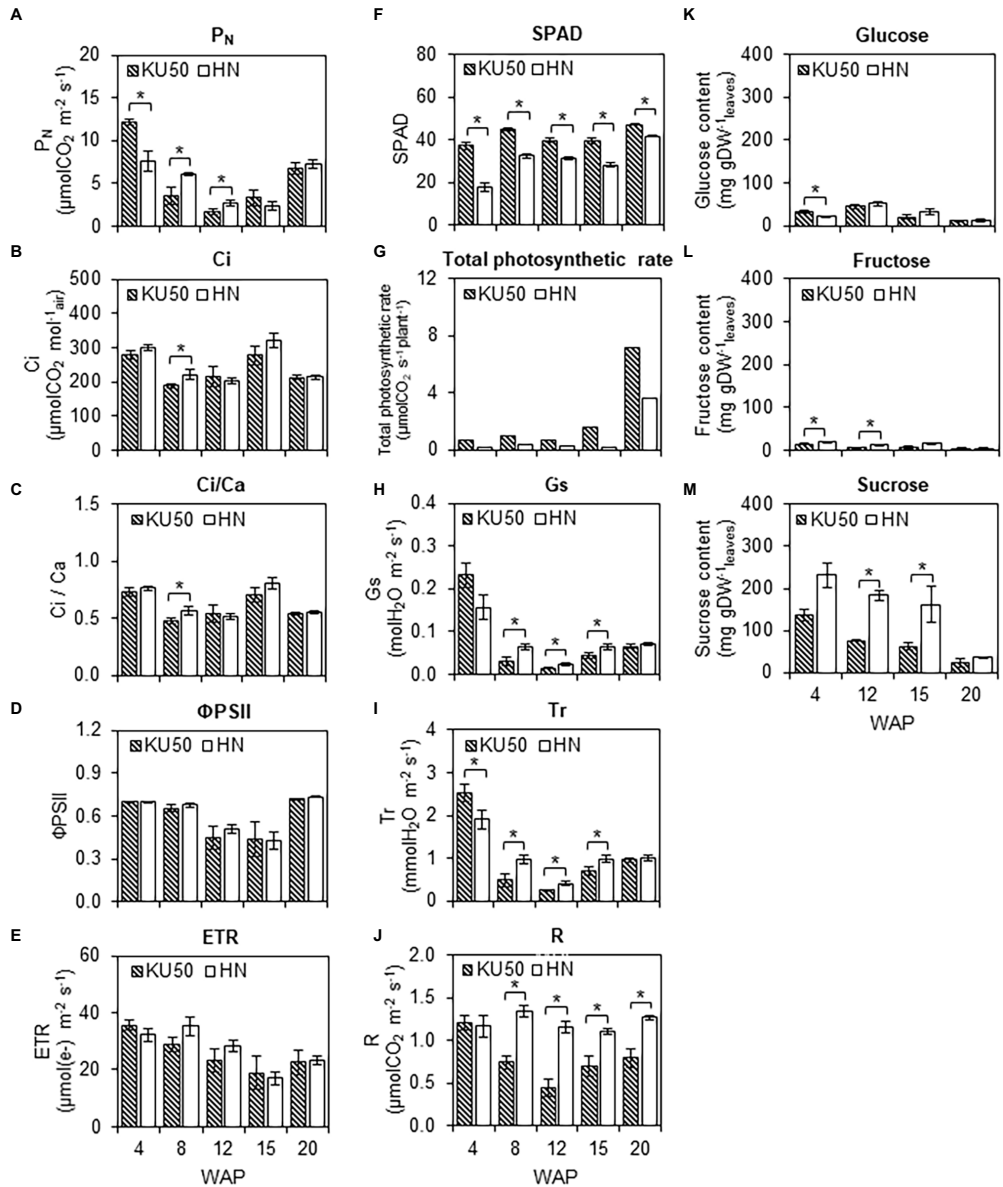
Photosynthesis capability and carbon assimilation of Kasetsart 50 (KU50) and Hanatee (HN) cassava varieties at various developmental stages. Leaf gas exchange and chlorophyll fluorescent parameters, including the **(A)** photosynthetic rate (P_N_), **(B)** intercellular CO_2_ concentration (Ci), **(C)** the ratio between intercellular and ambient CO_2_ concentrations (Ci/Ca), **(D)** effective quantum yield of photosystem II photochemistry (ΦPSII), **(E)** electron transport rate (ETR), **(F)** leaf greenness index (SPAD), **(G)** total photosynthetic rate, **(H)** stomatal conductance (Gs), **(I)** transpiration rate (Tr), **(J)** respiration rate (R), as well as soluble sugar contents, including **(K)** glucose, **(L)** fructose, and **(M)** sucrose, were measured from a fully expanded mature leaf at midday. Each result is the mean ± SE value obtained from five biological replicates. Statistical significance, based on a one-sided Student’s *t*-test, is denoted by ^*^p ≤ 0.05. The total photosynthetic rate was calculated by multiplying the average P_N_ value by the average total number of attached mature leaves for each variety. WAP, week after planting.

Differences in the growth and final yields of KU50 and HN were investigated based upon their individual genetic potential regarding photosynthesis and CO_2_ acquisition, carbon assimilation, and carbon allocation for root development. The photosynthetic capability was studied by measuring the leaf gas exchange (i.e., P_N_, Ci, Ci/Ca, Gs, Tr, and R), chlorophyll fluorescence (i.e., ΦPSII and ETR), and leaf greenness index (i.e., SPAD measurements; Figures 1A–J). The measurements were performed at the central lobe of the fully expanded first or the fourth leaf or the fifth leaf from the shoot apex because of its higher photosynthetic activity relative to others (Supplementary Figure S1A). Measurements were done using 1,000 μmol photons m^−2^ s^−1^, as this light intensity was sufficient to saturate the photosynthetic rates of the plants from this experiment (Supplementary Figure S1B). Figure 1A shows that P_N_ of KU50 declined from 12.21 ± 0.32 to 1.65 ± 0.33 μmol CO_2_ m^−2^ s^−1^ and HN declined from 7.58 ± 1.12 to 2.39 ± 0.33 μmol CO_2_ m^−2^ s^−1^ during the early stages of their development before increasing from 12 WAP for KU50 and 15 WAP for HN when fibrous roots transitioned into storage roots. Both varieties showed significantly different P_N_ (*p* ≤ 0.05) during canopy establishment (4–12 WAP) but leveled up thereafter. The P_N_ values measured during the experiment (4–20 WAP) were 1.65–12.21 μmol CO_2_ m^−2^ s^−1^ for KU50 and 2.39–7.58 μmol CO_2_ m^−2^ s^−1^ for HN (Figure 1A). The pattern of P_N_ were similar to that of the intercellular CO_2_ concentration (Ci), the ratio between intercellular and ambient CO_2_ concentrations (Ci/Ca), effective quantum yield of photosystem II (ΦPSII), and ETR, which showed that HN had slightly higher capability at the early development stage (8–15 WAP, Figures 1A–E). In contrast, KU50 maintained a significantly higher (*p* ≤ 0.05) leaf greenness index (SPAD) than HN throughout the experiment (Figure 1F). The results were corresponding to the greater chlorophyll content measured in KU50 leaves than in HN (Supplementary Figure S2). Of both varieties, KU50 had a higher total photosynthetic rate (reflecting overall photosynthetic capacity), calculated by multiplying the average P_N_ values by the total plant leaf area (Figure 1G). In addition, KU50 had lower stomatal conductance (Gs, Figure 1H) and transpiration rate (Tr, Figure 1I), consistent with its lower respiration rate (R, Figure 1J).

To investigate the carbon assimilation in cassava, three major soluble sugars, namely glucose, fructose, and sucrose, were measured in mature leaves during development. Results showed the sucrose content was higher than glucose and fructose, by at least 4-fold (Figures 1K–M). The leaf sucrose content of both varieties declined with plant age (Figure 1M). It was observed that HN leaves maintained a higher sucrose content throughout the developmental period (Figure 1M).

### Source-Sink Carbon Allocation in KU50 and HN

**Figure 2:**
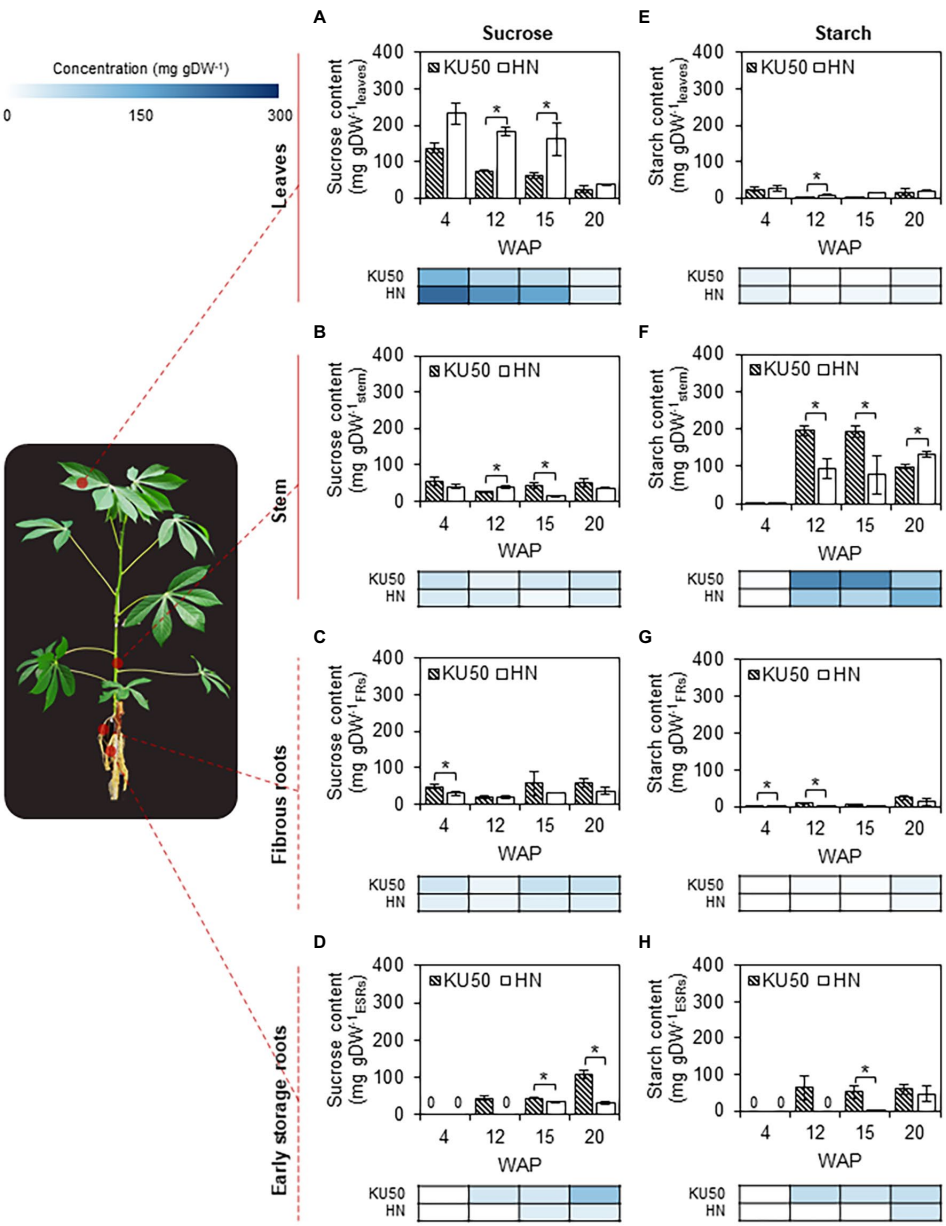
Changes in sucrose and starch contents in **(A,E)** leaves, **(B,F)** stems, **(C,G)** fibrous roots (FRs), and **(D,H)** early storage roots (ESRs) at various developmental stages of Kasetsart 50 (KU50) and Hanatee (HN) cassava varieties grown under greenhouse conditions, determined at midday on a dry weight basis. The heat map located under each figure clearly displays the proportion of each tissue on a dry weight basis at various developmental stages. Each result is the mean ± SE of values obtained from three biological replicates. Statistical significance, based on a one-sided Student’s *t*-test, is denoted by ^*^p ≤ 0.05. gDW, gram dry weight and WAP, week after planting.

Glucose, fructose, sucrose, and starch contents in shoot and root tissues of KU50 and HN were analyzed to investigate carbon allocation during plant development. Of these three major soluble sugars, sucrose was by far the most abundant in all the tissues at different developmental stages (Figure 2; Supplementary Figure S3). Therefore, we considered sucrose as a major form of carbon allocated for the growth of individual organs. The sucrose content in leaves slightly declined during the early stages of plant development and then decreased sharply after 15 weeks (Figure 2A). The results were observed in both cassava varieties, though HN had higher leaf sucrose content. KU50 showed sucrose content of 23.59–136.20 mg gram dry weight (gDW)^−1^_leaves_ compared to 37.18–232.48 mg gDW^−1^_leaves_ for HN during the experiment. Inversely, translocated sucrose contained in sink tissues was larger in KU50. The sucrose content in stems and FRs of both varieties was comparably low and leveled (Figures 2B,C), but the sucrose content in ESRs of KU50 was higher and tended to increase with plant age (Figure 2D). Overall, KU50 showed tissue sucrose contents of 25.98–54.36 mg gDW^−1^_stem_, 20.38–58.73 mg gDW^−1^_FRs_, and 44.89–108.43 mg gDW^−1^_ESRs_ compared to 14.71–40.52 mg gDW^−1^_stem_, 19.46–36.26 mg gDW^−1^_FRs_, and 32.82–33.85 mg gDW^−1^_ESRs_ for HN.

Starch content reflects the level of carbon accumulation in tissues. The transient pool of assimilated carbon stored in leaves as starch during daytime is later broken down to sugars for plant metabolism and partitioning to sink organs. KU50 and HN leaves contained smaller amounts of starch than sucrose, approximately 2.39–24.23 mg gDW^−1^_leaves_ in KU50 and 7.71–25.99 mg gDW^−1^_leaves_ in HN at 4–20 WAP (Figure 2E). On the contrary, starch content was higher in sink tissues, especially in the stem and storage roots (Figures 2F,H). KU50 accumulated more starch in its stem compared to HN at the early stage of plant development, and its starch content declined after storage root bulking (12–20 WAP). HN showed an opposite trend with higher starch accumulation in the stem at 20 WAP. In root tissues, starch was increasingly accumulated during root development. The profiles were more explicit in ESRs (Figures 2G,H). At the latter stage (20 WAP), the starch content in ESRs of KU50 (60.19 ± 14.70 mg gDW^−1^_ESRs_) was 1.29 times higher than that of HN (46.76 ± 21.46 mg gDW^−1^_ESRs_; Figure 2H).

**Figure 3:**
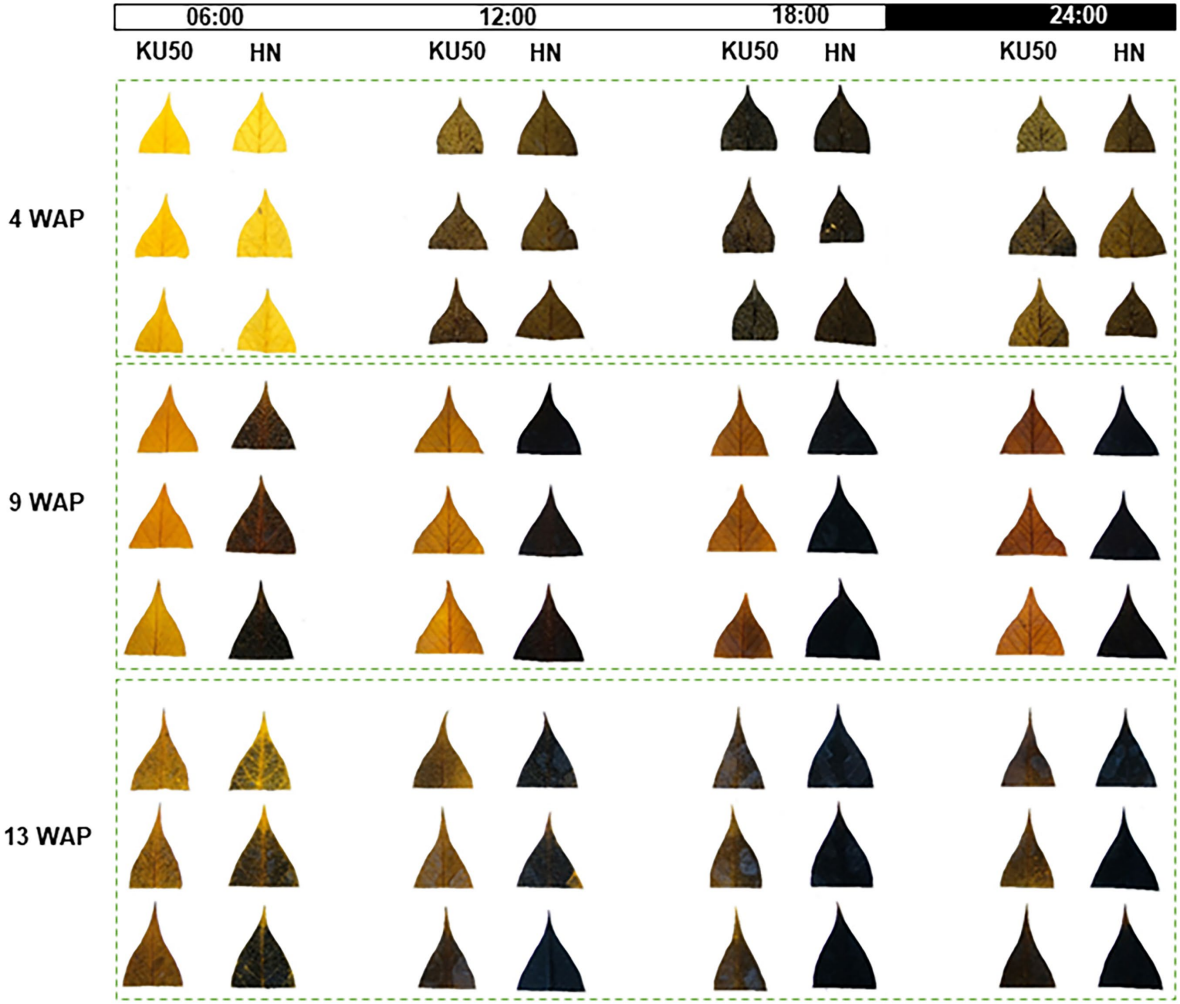
Iodine staining of the youngest fully expanded mature leaves of Kasetsart 50 (KU50) and Hanatee (HN) cassava varieties during 4–13 weeks after planting (WAP) in diurnal conditions.

**Figure 4:**
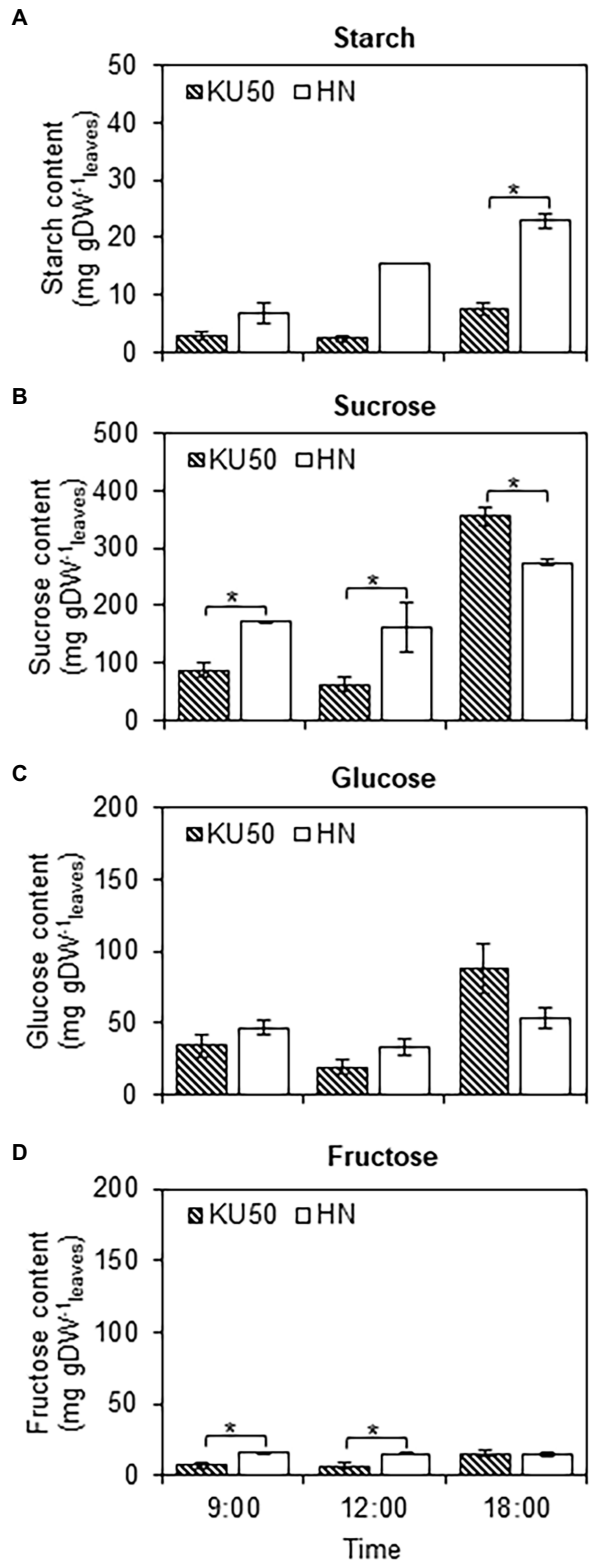
Changes in leaf starch **(A)**, sucrose **(B)**, glucose **(C)**, and fructose **(D)** contents of Kasetsart 50 (KU50) and Hanatee (HN) cassava varieties at 15 WAP in diurnal conditions, on a dry weight basis. Each result is the mean ± SE of values obtained from three biological replicates. Statistical significance, based on a one-sided Student’s *t*-test, is denoted by ^*^p ≤ 0.05. gDW, gram dry weight.

Interconversion between sucrose and starch in a day may affect their contents in plant tissues. Diurnal changes in carbon partitioning and allocation were investigated by monitoring the starch content in leaves during a 1-day cycle at different developmental stages. The dynamic accumulation of starch in leaves of KU50 and HN was inferred by iodine staining. The results showed diurnal changes in leaf starch content of both varieties during the 24-h cycle, ranging from the lowest at dawn (yellow-brown leaf) to the highest at dusk (dark blue leaf), followed by a decline during nighttime (Figure 3). At the early stage of plant development (4 WAP), both varieties showed a similar pattern of leaf starch content, increasing from the lowest accumulation at dawn (06:00) and peaking at dusk (18: 00; Figure 3). During storage root bulking (9–13 WAP), KU50 had a lower starch content and a clearer diurnal pattern than HN. Also, KU50 maintained a lower daytime leaf starch content than HN at 15 WAP (Figure 4A). Moreover, the soluble sugar analysis revealed KU50 had a significantly lower leaf sucrose content than HN after dawn and at midday comparing with the same plant age (15 WAP), but its leaf sucrose content was significantly higher at dusk (Figure 4B). The varietal differences in leaf glucose and fructose contents closely mirrored those of sucrose at 15 WAP (Figures 4C,D).

### Growth and Development of KU50 and HN in Greenhouse

**Figure 5:**
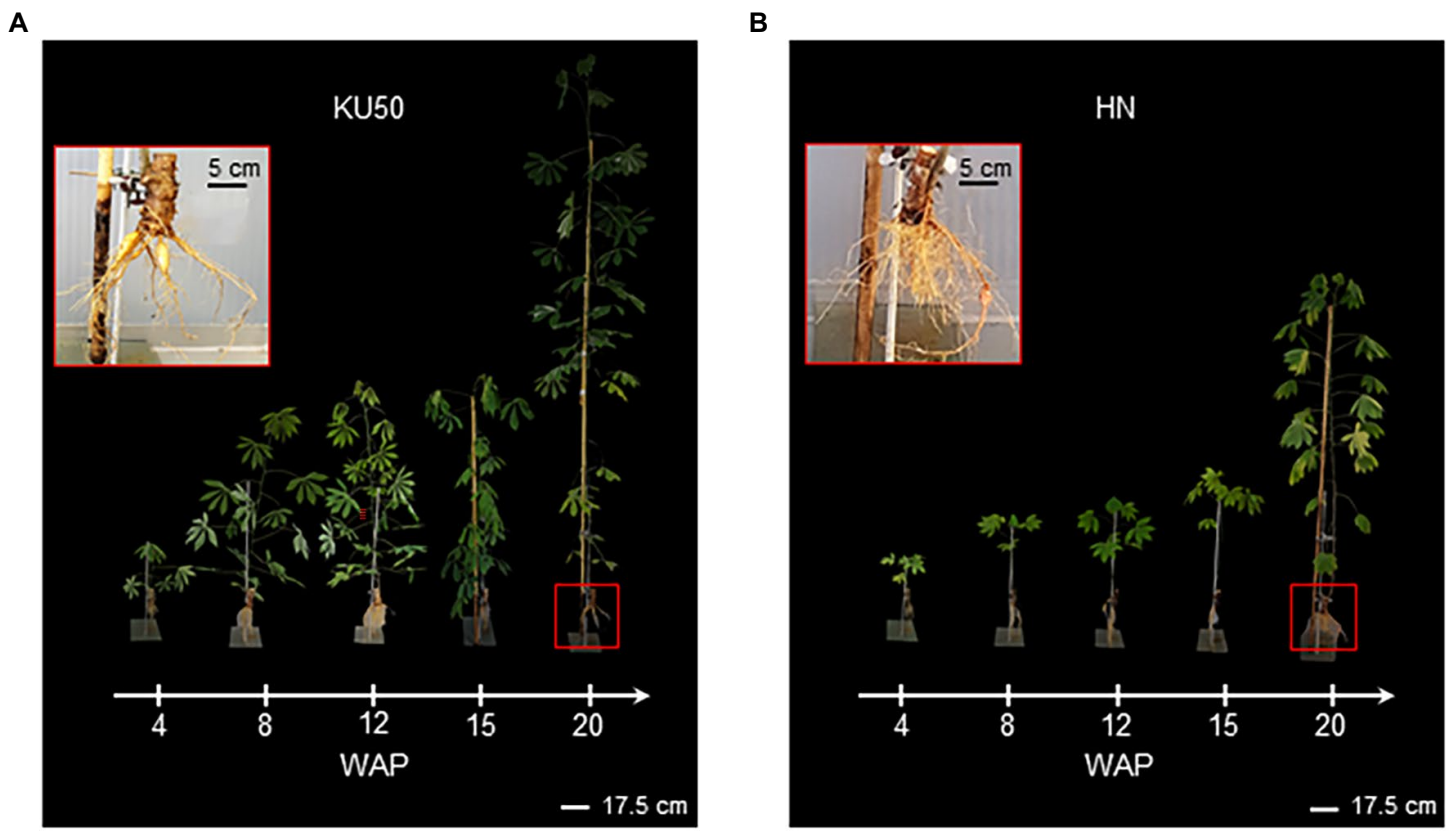
Growth and development of **(A)** KU50 and **(B)** HN cassava varieties grown under greenhouse conditions during 4–20 WAP. The top left panel shows the root structure of each cassava cultivar at 20 WAP.

Figure 5 showed that KU50 grew faster than HN and showed better shoot development and earlier storage root formation at the mature stage of plant development. It was observed that KU50 grew quickly after 4 weeks and then sharply at 15 WAP (Figure 5A), whereas HN gradually grew up to 15 WAP before drastically increasing (Figure 5B). KU50 had a better-developed leaf canopy with a greater number of photosynthetic leaves than HN at the same plant age. At 20 WAP when all samples were finally harvested, KU50 had more storage roots than HN (Figure 5; the top left panel). Furthermore, the noninvasive MRI showed the root system of KU50 was better developed (Supplementary Figure S4), corresponding to its higher storage root yield.

**Figure 6:**
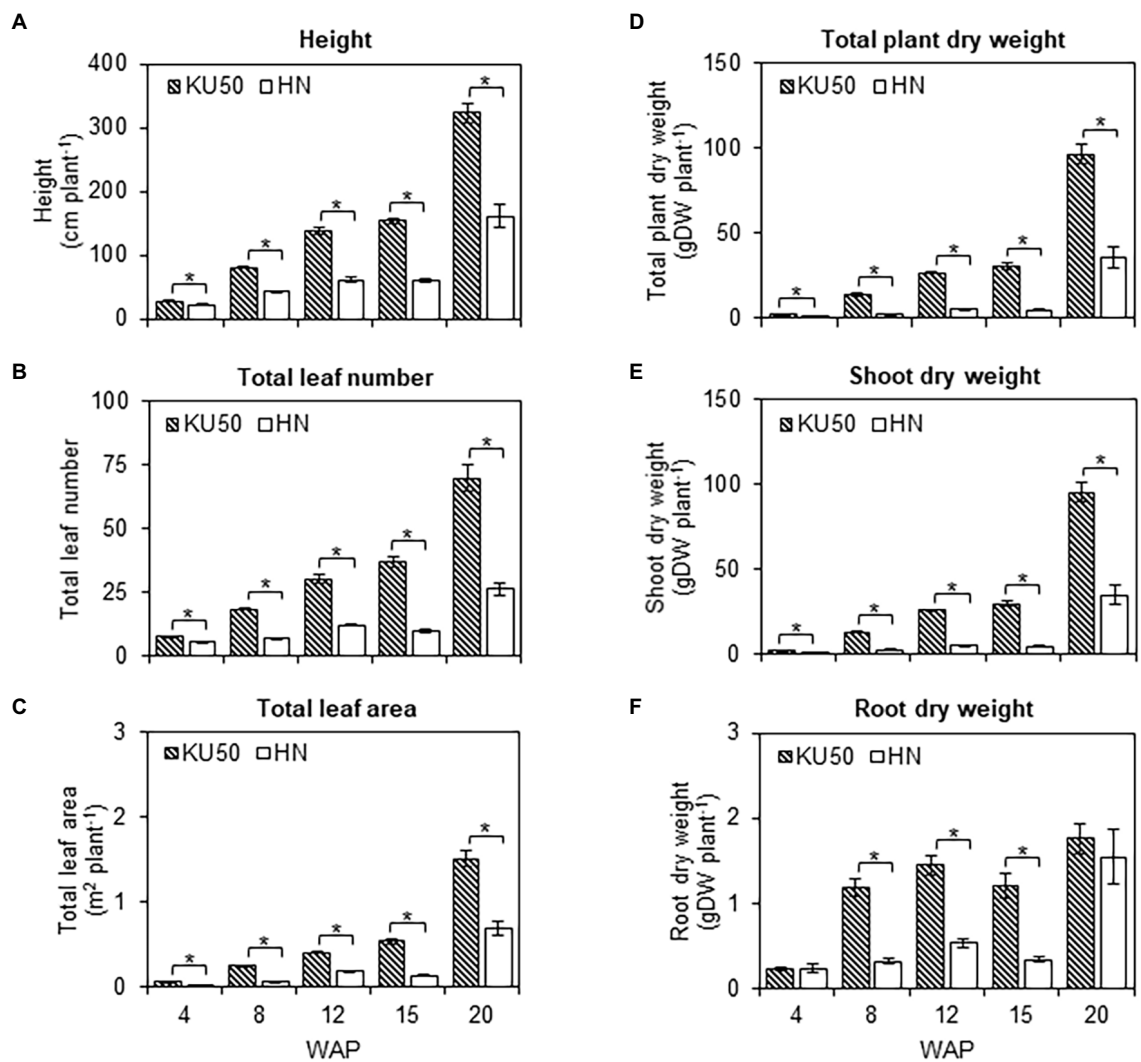
**(A)** Height, **(B)** total leaf number, **(C)** total leaf area, **(D)** total plant dry weight, **(E)** shoot dry weight, and **(F)** root dry weight of KU50 and HN cassava varieties at various developmental stages. Each result is the mean ± SE of values obtained from 15 biological replicates at 4, 8, and 12 WAP, and from 12 biological replicates at 15 and 20 WAP. Statistical significance, based on a one-sided Student’s *t*-test, is denoted by ^*^p ≤ 0.05. gDW, gram dry weight.

Analysis of plant growth and shoot-root development revealed differences in the carbon partitioning strategies of both varieties (Figure 6). KU50 was significantly taller than HN across the developmental stages (Student’s *t*-test, *p* ≤ 0.05; Figure 6A) and was twice the height of HN at 20 WAP (Figure 6A). The total leaf number and leaf area closely followed a similar pattern to the plant height, with KU50 having approximately 2.69- and 2.17-times higher values at 20 WAP, respectively (Figures 6B,C). For biomass measurement, the total plant dry weight of KU50 was significantly higher than that of HN at the different stages of development and approximately 2.67 times greater than HN at 20 WAP (Figure 6D). The increase in plant biomass was mainly from shoot development—i.e., leaves, petioles, and stems, rather than roots—i.e., FRs and ESRs (Figures 6E,F). Of the two varieties, the shoot dry weight of KU50 was significantly higher across the development stages, higher by as much as approximately 2.74 times at 20 WAP (Figure 6E). During the experiment (4–20 WAP), the plants actively developed leaves and stems to boost light interception for photosynthesis and carbon assimilation to provide sufficient substrates for storage root formation. The total root dry matter was significantly greater in KU50 than HN for 8–15 WAP in our experiment (Figure 6F), which does not reflect the entire growth period from 6 to 12 months in commercial productions. At maturity (20 WAP), KU50 roughly measured 322.63 ± 14.87 cm in height with 70 ± 5 leaves plant^−1^ averaging 1.50 ± 0.09 m^2^ leaf area plant^−1^, and 96.73 ± 5.67 gDW plant^−1^ for the total dry biomass (94.97 ± 5.59 gDW plant^−1^ for the shoot and 1.76 ± 0.17 gDW plant^−1^ for total roots); HN measured 160.79 ± 17.34 cm in height with 26 ± 2 leaves plant^−1^ averaging 0.69 ± 0.09 m^2^ leaf area plant^−1^, and 36.23 ± 6.26 gDW plant^−1^ for the total dry biomass (34.68 ± 5.97 gDW plant^−1^ for the shoot and 1.55 ± 0.32 gDW plant^−1^ for total roots; Figure 6).

**Figure 7:**
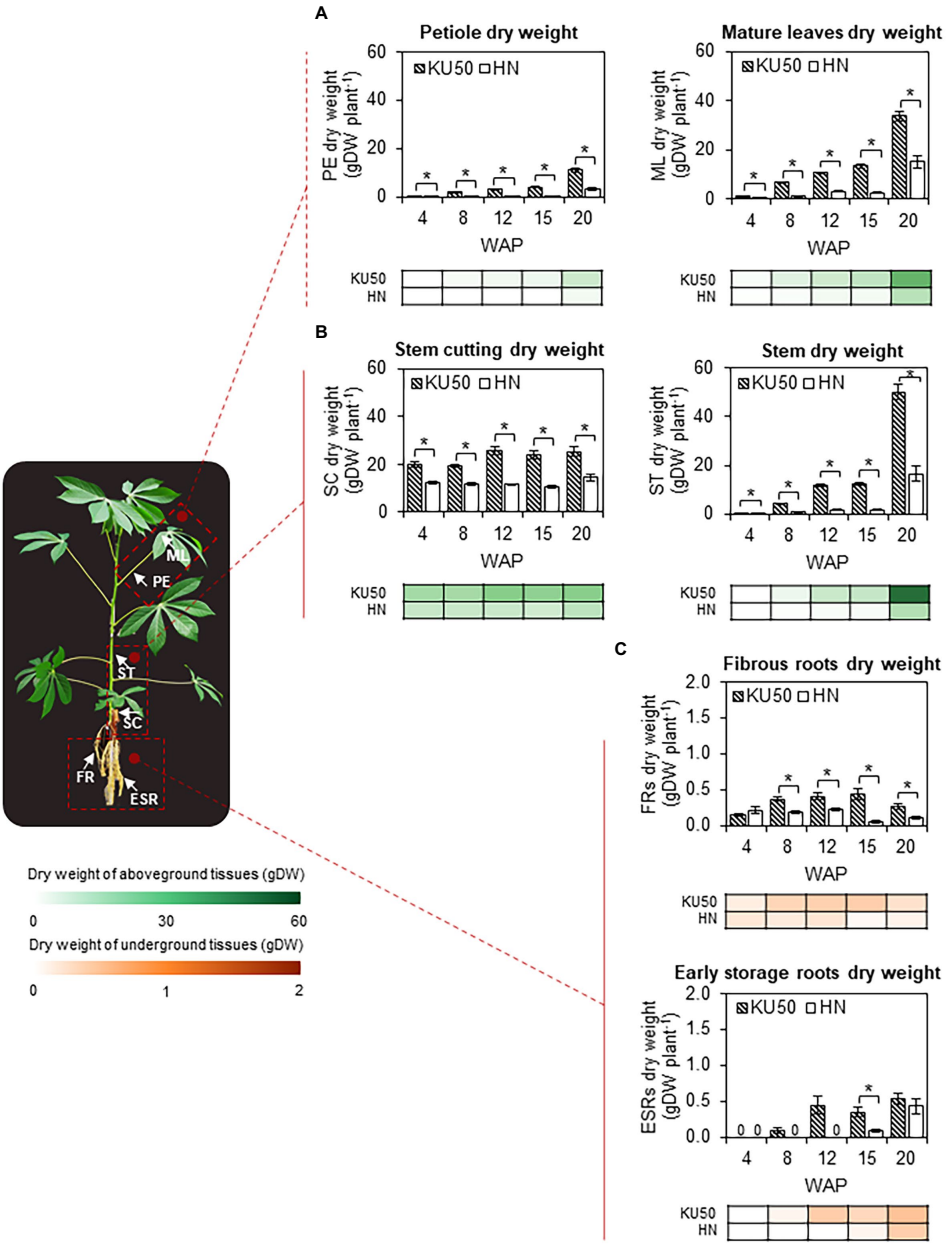
Dry matter accumulation in **(A)** petioles (PE) and mature leaves (ML), **(B)** stem cuttings (SC) and stems (ST), and **(C)** fibrous adventitious roots (FRs) and ESRs of KU50 and HN cassava varieties at various developmental stages. The heat map located under each figure clearly displays the proportion of each tissue on a dry weight basis at various developmental stages. Each result, excepting the dry weight of ESRs, is the mean ± SE of values obtained from 15 biological replicates at 4, 8, and 12 WAP, and from 12 biological replicates at 15 and 20 WAP. Data on the ESRs dry weight are the mean ± SE of values obtained from three biological replicates at different stages of development. Statistical significance, based on a one-sided Student’s *t*-test, is denoted by ^*^p ≤ 0.05. gDW, gram dry weight.

The varietal differences in shoot-root carbon partitioning were investigated by studying the biomass accumulation patterns of the individual plant tissues (Figure 7), namely shoots, separated into petioles, mature leaves, and stems, roots, divided into FRs and ESRs, and stem cuttings. KU50 maintained significantly (*p* ≤ 0.05) higher dry weights of petioles and mature leaves than HN throughout the studied periods, indicating better-developed photosynthetic tissues (Figure 7A). The petiole and mature leaf dry weights of both varieties increased steadily until 15 WAP and then steeply afterward, with KU50 having approximately 3.62 and approximately 2.23 times higher values at 20 WAP, respectively (Figure 7A). Similarly, KU50 showed greater stem development, maintaining significantly higher stem dry weight (*p* ≤ 0.05) all through the developmental stages and outperforming HN by approximately 3.04 times at 20 WAP (Figure 7B; right). At 20 WAP, the petiole, mature leaf, and stem dry weights of KU50 were 11.00 ± 0.70, 33.76 ± 2.04, and 50.22 ± 3.23 gDW plant^−1^, respectively. By comparison, HN had a petiole dry weight of 3.04 ± 0.52 gDW plant^−1^, mature leaf dry weight of 15.11 ± 2.36 gDW plant^−1^, and stem dry weight of 16.54 ± 3.12 gDW plant^−1^. For underground biomass, KU50 showed greater FRs development from 8 WAP onward (Figure 7C; top). Varietal differences in storage root bulking were observed. While the conversion from fibrous roots to storage roots began as early as 8 WAP, it took about 15 WAP till ESRs were first observed in HN. At 20 WAP, KU50 had 0.26 ± 0.04 gDW of FRs plant^−1^ and 0.53 ± 0.08 gDW of ESRs plant^−1^, while HN had a 0.11 ± 0.03 gDW of FRs plant^−1^ and 0.43 ± 0.11 gDW of ESRs plant^−1^ (Figure 7C). It is worth noting that stem cuttings of both cultivars showed an increase in dry matter accumulation after propagation until storage root bulking, *ca.* 8 WAP for KU50 and 15 WAP for HN (Figure 7B; left).

## Discussion

Crop yield improvement is a global agenda to avoid food insecurity in the future. To achieve this, research has been focused on elevating physiological characteristics related to (i) the efficiency of crops to intercept radiation (photosynthetic capability), (ii) the efficiency of intercepted radiation conversion into biomass (carbon assimilation), and (iii) the efficiency of biomass partitioning into the harvested product (carbon allocation; Long et al., 2006). Studies have shown that the photosynthetic capability of cassava genotypes does not vary as much as the storage root yields (Mahon et al., 1977; El-Sharkawy et al., 1984; Edwards et al., 1990; El-Sharkawy and Cock, 1990). Photosynthetic capability determines carbon assimilation and source-to-sink carbon allocation, proposed as central to growth and storage root development of cassava, and is under genetic and environmental control.

### Shoot-to-Root Carbon Partitioning Modulated Root Yield in KU50 and HN

**Figure 8:**
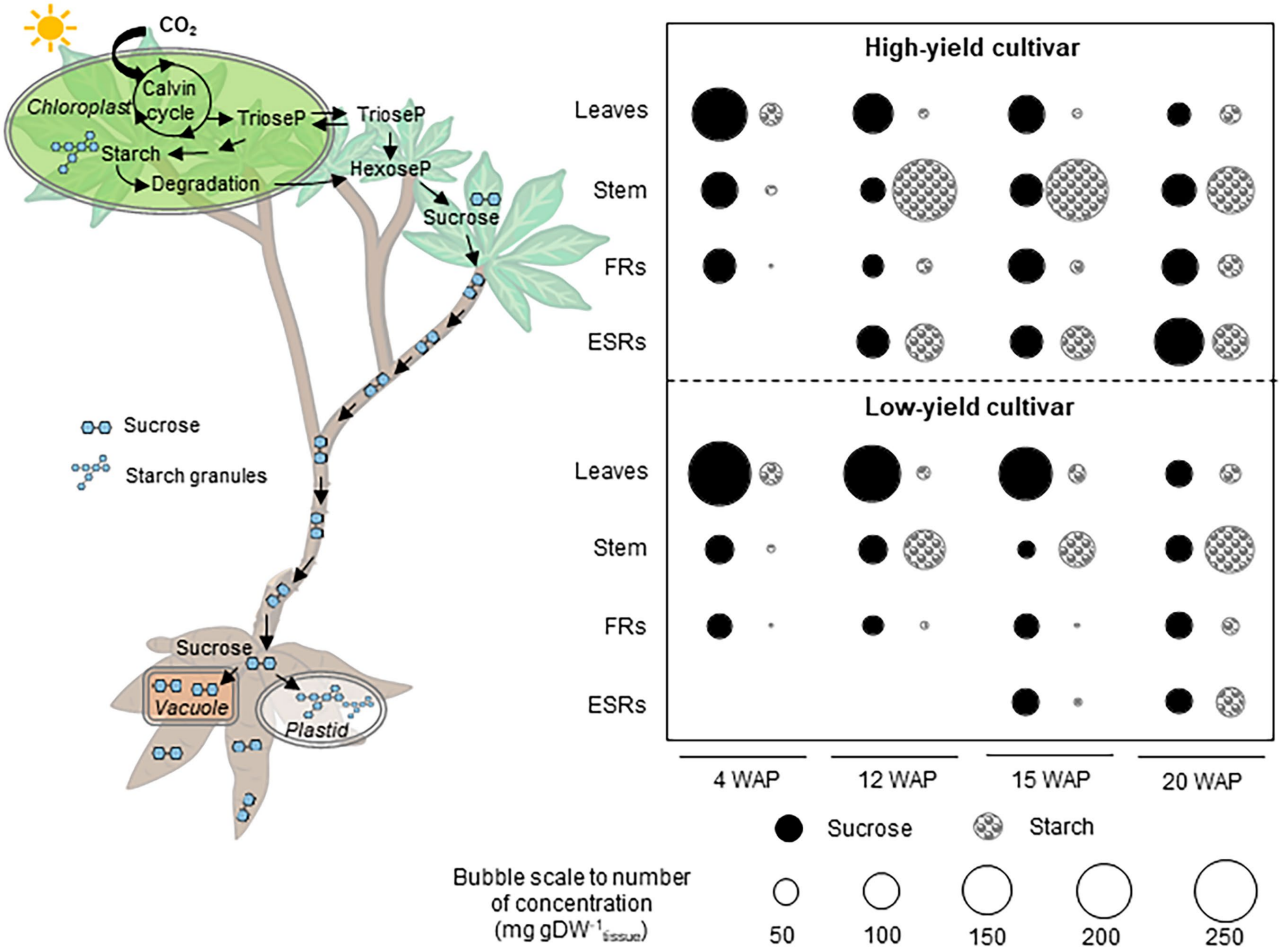
Scheme summarizing the shoot-to-root carbon partitioning in high- and low-yield cassava varieties at various developmental stages. The partitioning of carbon into shoots and roots was determined from changes in sucrose and starch contents. ESRs, early storage roots; FRs, fibrous roots; and WAP, week after planting.

Photosynthetic capacity, carbon assimilation, and source-sink carbon allocation were demonstrated to be key factors underlying the high root yield of KU50. Under similar experimental conditions, KU50 proved genetically superior to HN in relation to the photosynthetic capacity, carbon assimilation, and source-sink carbon allocation toward root development. Here, sucrose and starch accumulation in the source (leaves) and sink (stem, FRs, and ESRs) tissues during cassava plant development were analyzed to study the patterns of carbon allocation, also referred to as shoot-root carbon partitioning, in these distinct varieties (Figure 8). The investigation captured different stages of development from sprouting to canopy establishment approximately 4–20 WAP when cassava plants tend to highly develop their shoot, including the storage root bulking stage (after 8 WAP) when massive carbon is mobilized for starchy root growth. Considering the patterns of sucrose and starch accumulation in the individual plant tissues, both varieties had a similar profile across the sampling dates, which indicated the association of carbon assimilation and allocation to the developmental stages. The leaf sucrose level tended to decrease with age, while the sucrose content in the stem and FRs seemed to be constant. In contrast, the ESRs showed an increasing sucrose content with age (Figure 8). Moreover, the leaf starch content seemed stable across all sampling dates, whereas the pattern of starch in stems, FRs, and ESRs tended to increase during cassava plant development (Figure 8).

Differences in pool sizes of the carbon substrates were observed between the varieties, which may reflect varying carbon assimilation and allocation capacity linked to their genetic backgrounds. Varietal differences in patterns of sucrose and starch accumulation in shoot and root tissues were found from 12th WAP. The patterns observed in KU50 plants at 12 WAP were similar to those in HN at 15 WAP when the storage roots were first observed. These patterns likely demonstrated the carbon allocation profile at the root bulking stage of cassava development. Taken together, the high-yielding KU50 showed decreasing leaf sucrose and increasing root sucrose during 4–20 WAP, with fairly even source-sink (leaf-root) sucrose distribution at 15 WAP. KU50 accumulated the least amount of starch in all tissues, except leaves, at 4 WAP and the most in stems during storage root bulking (12–15 WAP), which tended to be increasingly remobilized for storage root growth (Figure 8; top). For the low-yielding HN, tissue-accumulated sucrose decreased from leaves to roots during 4–15 WAP, with an even source-sink distribution at 20 WAP. Little accumulated starch was found in each plant tissue at 4 WAP compared to sucrose, and the highest content of starch was accumulated in stems during bulking of storage roots (Figure 8; bottom). The relatively high sucrose content in leaves of HN may reflect its low capability to allocate carbon for supporting the growth of root systems. During storage root bulking (15–20 WAP), the low-yielding variety seemed to accumulate more starch to its stem than to roots, unlike the high-yielding variety, which increasingly allocated more starch to roots (Figure 8). In summary, the results shed light on the role of shoot-root carbon allocation in modulating the final root yield of cassava plants and showed that this characteristic is associated with the genetic background of individual varieties (Supplementary Figure S5; Mahakosee et al., 2019; Phoncharoen et al., 2019).

Overall, we report different patterns of sucrose and starch accumulation in source and sink tissues of a low-yielding landrace (HN) and a modern high-yielding variety (KU50) linking to their growth. Both varieties exhibited distinct strategies for carbon assimilation and allocation to shoots and roots (Figures 2, 8). KU50 maintained a lower level of sucrose in leaves and tended to allocate more carbon substrates to stems and storage roots. The superior shoot-to-root carbon allocation effectiveness of KU50 was demonstrated by its higher starch accumulation in sink organs (Figures 2, 8; top) and a higher diurnal pattern of sucrose and starch interconversion in leaves (Figures 3, 4). It is also worth noting that at the starch filling stage, after root bulking, KU50 tended to allocate carbon to developing storage roots rather than stems (Figures 2, 8; top), which differs from the allocation strategy of HN. For the low-yielding variety HN, its higher leaf sucrose content may not be a result of a superior photosynthetic rate but rather indicative of inefficiency in carbon allocation to sink organs due to its genetic background. HN slowly allocated carbon to stems and storage roots. The increase in leaf starch content observed in both KU50 and HN during the transition to root bulking may reflect a higher level of carbon assimilation required to support storage root formation and root starch filling. A previous study showed differences in routes of carbon utilization in storage roots of both KU50 and HN cassava, with KU50 requiring high carbon for synthesizing carbohydrates and amino acids and for use as a precursor for biomass production, through constraint-based metabolic modeling (Chiewchankaset et al., 2019). Similar patterns of source-to-sink carbon allocation were found in Huanan 124 and Fuxuan 01, which are high- and low-yielding varieties, respectively (Li et al., 2016). These results demonstrate that carbon assimilation and allocation are genetic traits strongly associated with plant growth and storage root yield of cassava. Our findings open a promising opportunity for exploring carbon partitioning and utilization to improve high-yielding cassava genotypes in crop improvement programs.

### Growth and Development of KU50 and HN in Greenhouse

Kasetsart 50 plants grew well with higher storage root yield than HN under a controlled environment, (Figures 6F, 7C; bottom), according to its photosynthesis capability (Figure 1A). Our study showed that plant growth and root yield were highly associated with photosynthetic capacity (Figures 1G, 5–7). This result corresponds to several studies, which show a strong positive correlation between leaf characteristics (i.e., total leaf area, leaf area index, and leaf area duration) and storage root yield (e.g., Cock, 1976; El-Sharkawy, 2007; Pipatsitee et al., 2019). HN had higher photosynthetic characteristics (i.e., P_N_, Ci, Ci/Ca, ΦPSII, ETR, Gs, and Tr) than KU50 (Figures 1A–E,H,I), which may reflect a typical landrace trait as suggested by Panda et al. (2018) and Mathan et al. (2021). Similar findings were reported by De Souza and Long (2018), who compared the light saturated P_N_ of genetically improved cassava varieties TMS 98/0581 and TMS 30572 with the TME 7 and TME 419 landraces. Although HN had a high photosynthetic capability, it showed a higher metabolic cost as revealed by the greater leaf respiration rate (Figure 1J). Respiration is involved in the carbon catabolic process that metabolizes carbon substrates to produce high energy molecules for fueling the entire metabolism. There is a reciprocal relationship between the respiration level and cellular biomass biosynthesis (Collalti et al., 2020). Hurry et al. (2005) showed a decrease in plant dry matter production under intensive respiration. The results may explain the low root yield of variety HN. Interestingly, it was observed that P_N_ of HN and KU50 varied across the developmental stages, exhibiting a V-shaped pattern (Figure 1A). This pattern was also observed in other cassava varieties, including Rayong 9 (Vongcharoen et al., 2018; Santanoo et al., 2020), Rayong 11, CMR38-125-77 (Santanoo et al., 2020), MBra 110, MMal 48, MCol 22, and MPan 51 (El-Sharkawy and De Tafur, 2010), indicating a characteristic of cassava species. The results indicate a strong interconnection between photosynthesis capacity and intracellular carbon metabolism and allocation.

## Conclusion

The carbon assimilation in plant metabolism and source-to-sink carbon allocation are believed to be key modulators of plant growth and the final root yield of cassava. These characteristics have been highly linked to the genetic potential of the individual varieties. Our study reveals that the high root yield of cassava is related to the photosynthetic capacity, which is dependent on P_N_ and plant leaf traits rather than the photosynthetic capability. Though, improvement of photosynthetic rate along with other complementary traits can boost crop yield. Furthermore, the modern high-yielding variety (KU50) and the low-yielding landrace (HN) showed different patterns of carbon assimilation and shoot-to-root carbon allocation. KU50 proved superior in allocating carbon from source to sink organs for their growth, while HN allocated photoassimilates more to stems than storage roots and showed a higher metabolic cost in terms of respiration. The knowledge gained from this study may be useful for whole plant constraint-based metabolic modeling and may be used as a criterion for screening and selecting high-yield genotypes in cassava breeding programs.

## Data Availability Statement

The original contributions presented in the study are included in the article/Supplementary Material, further inquiries can be directed to the corresponding author.

## Author Contributions

This study was conceived and designed by TS and TW. TS, SK, and TW supervised the project. PC cultivated plants, collected samples, and performed plant growth analysis and physiological measurement. JT, PB, and PC performed biochemical analysis. TS and PC analyzed all data and interpreted the results and drafted the manuscript. TS, PC, JT, SK, and TW discussed the results. All authors interpreted the results, wrote the article, and approved the final manuscript.

## Funding

This work was financially supported by National Science and Technology Development Agency (NSTDA), Thailand (grant no. P-16-50362; CASSAVASTORe project) and the National Science Research and Innovation Fund (NSRF) Thailand (grant no. 42951). Also, we would like to thank the Postdoctoral Fellowship from King Mongkut’s University of Technology Thonburi (KMUTT), Thailand for PC; and the Postdoctoral Fellowship from the Program Management Unit for Human Resources and Institutional Development, Research, and Innovation (grant no. B01F630003) for PB.

## Conflict of Interest

The authors declare that the research was conducted in the absence of any commercial or financial relationships that could be construed as a potential conflict of interest.

## Publisher’s Note

All claims expressed in this article are solely those of the authors and do not necessarily represent those of their affiliated organizations, or those of the publisher, the editors and the reviewers. Any product that may be evaluated in this article, or claim that may be made by its manufacturer, is not guaranteed or endorsed by the publisher.
