# Supplementary material for: Effective Metabolic Carbon Utilization and Shoot-to-Root Partitioning Modulate Distinctive Yield in High Yielding Cassava Variety
Source: Front Plant Sci. 2022 Feb 16;13:832304. doi: 10.3389/fpls.2022.832304 (PMC8888839; doi:10.3389/fpls.2022.832304)
Supplement: Supplementary file 1 [file Data_Sheet_1.PDF]

Supplementary Material

1 Supplementary Figures

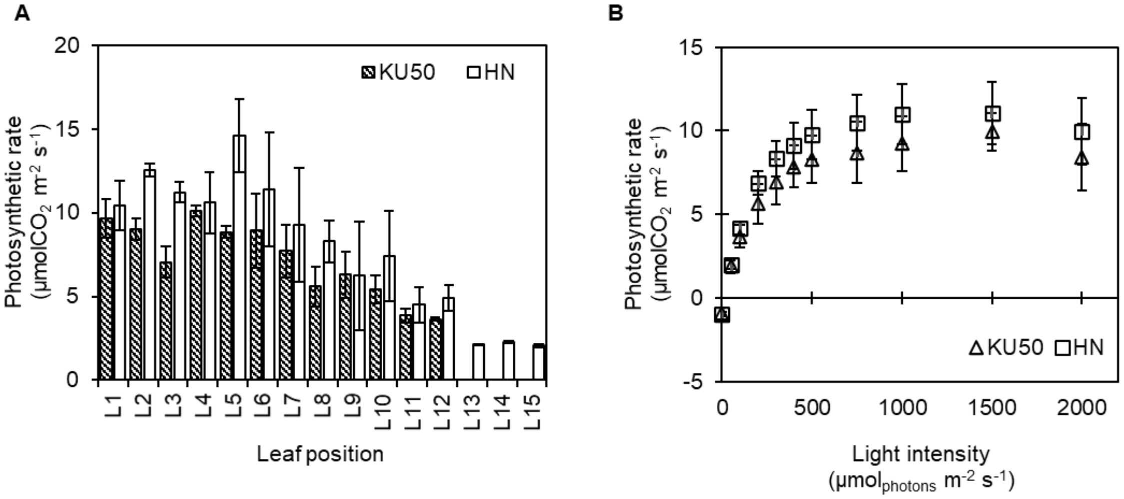

Supplementary Figure S1 Photosynthetic rates of Kasetsart 50 (KU50) and Hanatee (HN) cassava varieties grown under greenhouse conditions at 14-15 weeks after planting, measured **(A)** at different developmental stages of leaves at the light intensity of 1,000 µmol photons m^{-2} s^{-1} and **(B)** from the youngest fully expanded cassava leaf at the light intensity of 0-2,000 µmol photons m^{-2} s^{-1}. Each result is the mean ± SE of values obtained from three biological replicates.

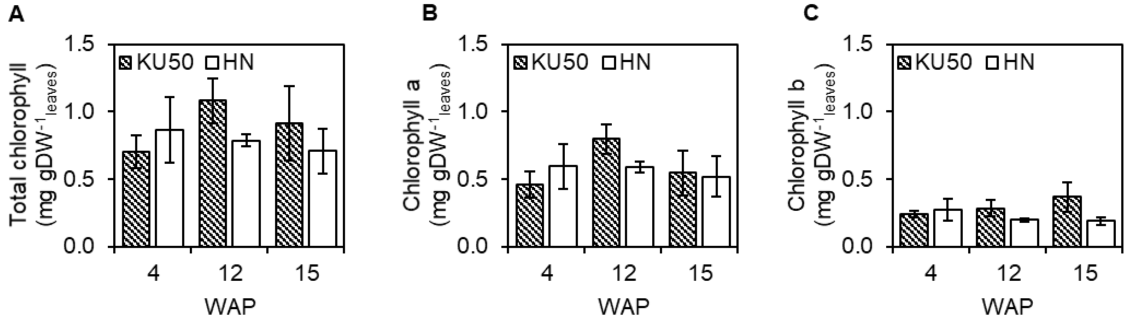

Supplementary Figure S2 (A) Total chlorophyll, **(B)** chlorophyll a, and **(C)** chlorophyll b of Kasetsart 50 (KU50) and Hanatee (HN) cassava varieties grown under greenhouse conditions. Each result is the mean ± SE of values obtained from three biological replicates. Statistical significance, based on a one-sided Student's t-test, is denoted by * ($p \leq 0.05$). All results are not statistically significant. gDW = gram dry weight and WAP = week after planting.

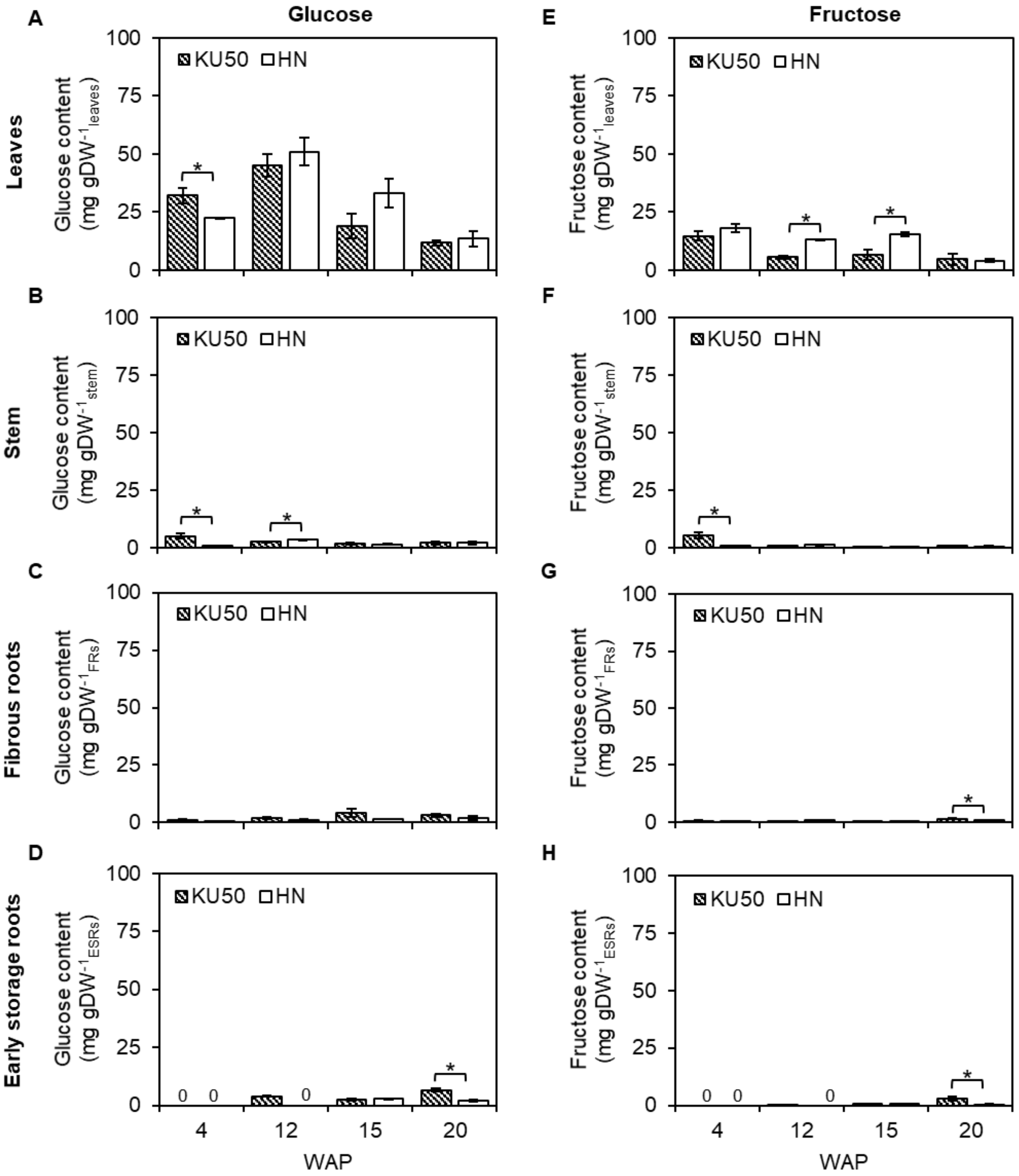

Supplementary Figure S3 Changes in glucose and fructose contents of leaves **(A, E)**, stems **(B, F)**, fibrous roots (FRs) **(C, G)**, and early storage roots (ESRs) **(D, H)** of Kasetsart 50 (KU50) and Hanatee (HN) cassava varieties at various developmental stages, determined at midday on a dry weight basis. Each result is the mean ± SE of values obtained from three biological replicates. Statistical significance, based on a one-sided Student's t-test, is denoted by * ($p \leq 0.05$). gDW = gram dry weight and WAP = week after planting.

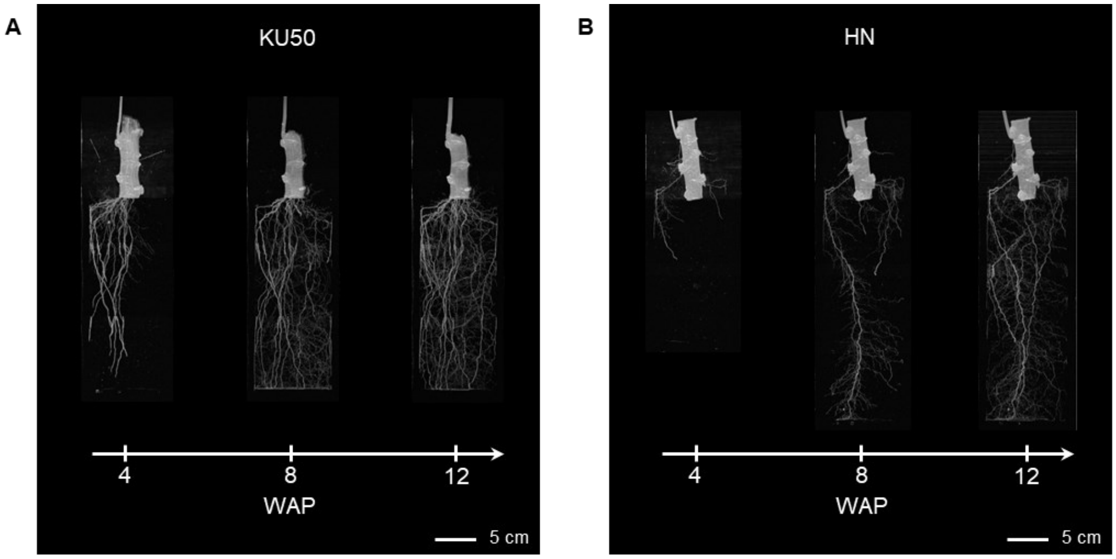

Supplementary Figure S4 The development of root systems of **(A)** Kasetsart 50 (KU50) and **(B)** Hanatee (HN) cassava varieties captured by Magnetic Resonance Imaging, a noninvasive method, during 4-12 weeks after planting (WAP) under greenhouse conditions.

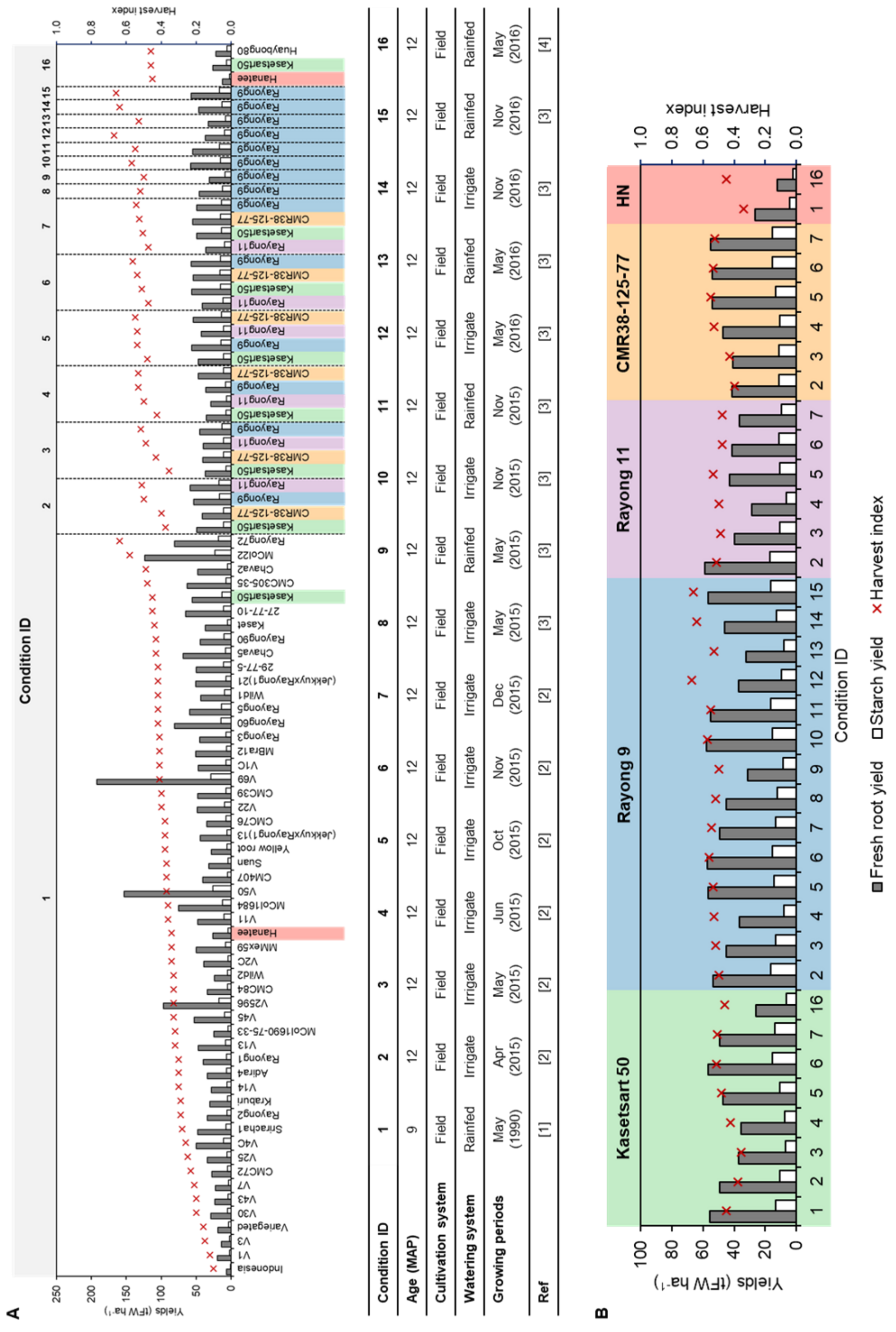

Supplementary Figure S5 Changes in fresh root yield, root starch yield, and harvest index of **(A)** different cassava genotypes grown under the same growing conditions and **(B)** similar genotypes grown in different growing conditions. The results demonstrated the association of these characteristics predominantly to genetic background of individual varieties. The genetically improved varieties have greater root yield, root starch content, and harvest index with respect to cassava landrace variety. Data were collected from literature, including Boonseng et al., 1999 [1], Phoncharoen et al., 2019 [2],

Mahakosee et al., 2019 [3], and Chaengsee et al., 2020 [4]. Each color represents each of the cassava genotypes, green for Kasetsart 50, blue for Rayong 9, purple for Rayong 11, orange for CMR38-125-77, and red for Hanatee (HN).
